# Publisher Correction: Disproportionate exposure to urban heat island intensity across major US cities

**DOI:** 10.1038/s41467-021-23972-6

**Published:** 2021-06-28

**Authors:** Angel Hsu, Glenn Sheriff, Tirthankar Chakraborty, Diego Manya

**Affiliations:** 1 Yale-NUS College, Singapore, Singapore; 2 School of Public Policy, University of North Carolina at Chapel Hill, Chapel Hill, NC USA; 3 Data-Driven EnviroLab, Singapore, Singapore; 4 School of Politics and Global Studies, Arizona State University, Tempe, AZ USA; 5 School of the Environment, Yale University, New Haven, CT USA

**Keywords:** Climate-change impacts, Environmental social sciences, Environmental impact, Environmental health

Correction to: *Nature Communications* 10.1038/s41467-021-22799-5, published online 25 May 2021.

The original version of this Article contained an error in Fig. 2, in which a legend has been missing. This has been corrected in both the PDF and HTML versions of the Article.

The original version of the Peer Review File associated with this Article was updated shortly after publication to redact private notes from the authors.

## Supplementary information

Peer Review File

