# Supplementary material for: Disproportionate exposure to urban heat island intensity across major US cities
Source: Nat Commun. 2021 May 25;12:2721. doi: 10.1038/s41467-021-22799-5 (PMC8149665; doi:10.1038/s41467-021-22799-5)
Supplement: Supplementary file 1 — Supplementary Information [file 41467_2021_22799_MOESM1_ESM.pdf]

Disproportionate exposure to Urban Heat Island across major U.S. cities

Angel Hsu, Glenn Sheriff[a], Tirthankar Chakraborty and Diego Manya

March 11, 2021

[a]Corresponding author. Email: gsheriff@asu.edu

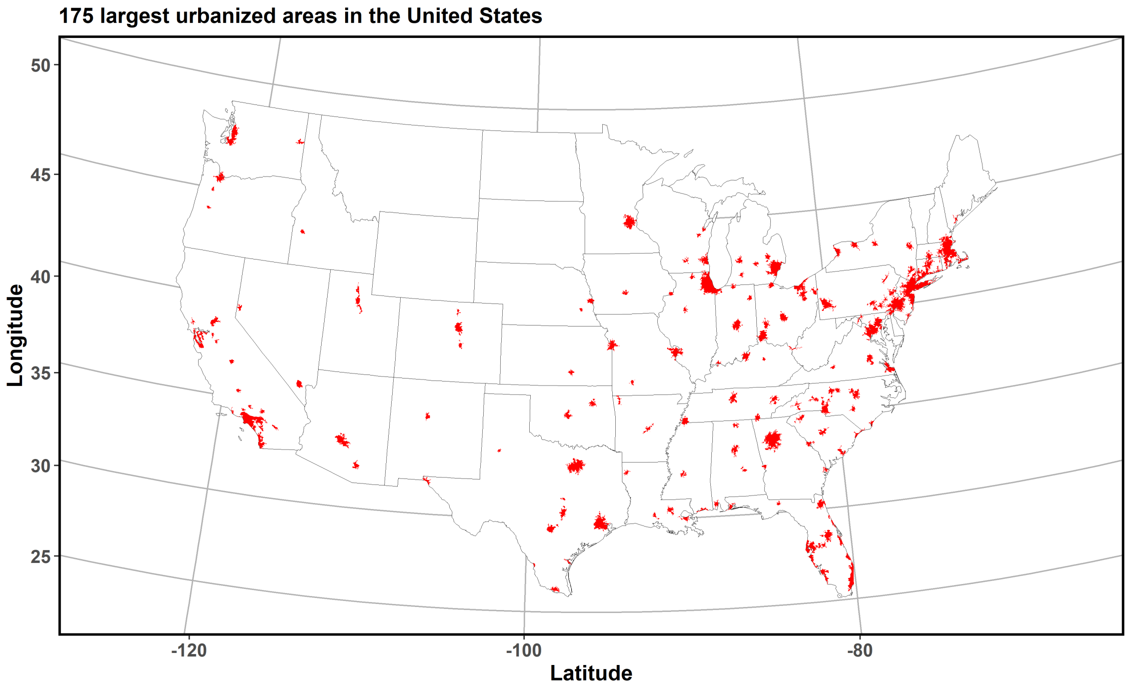

Supplementary Figure 1. Location and extent of urbanized areas evaluated in this study

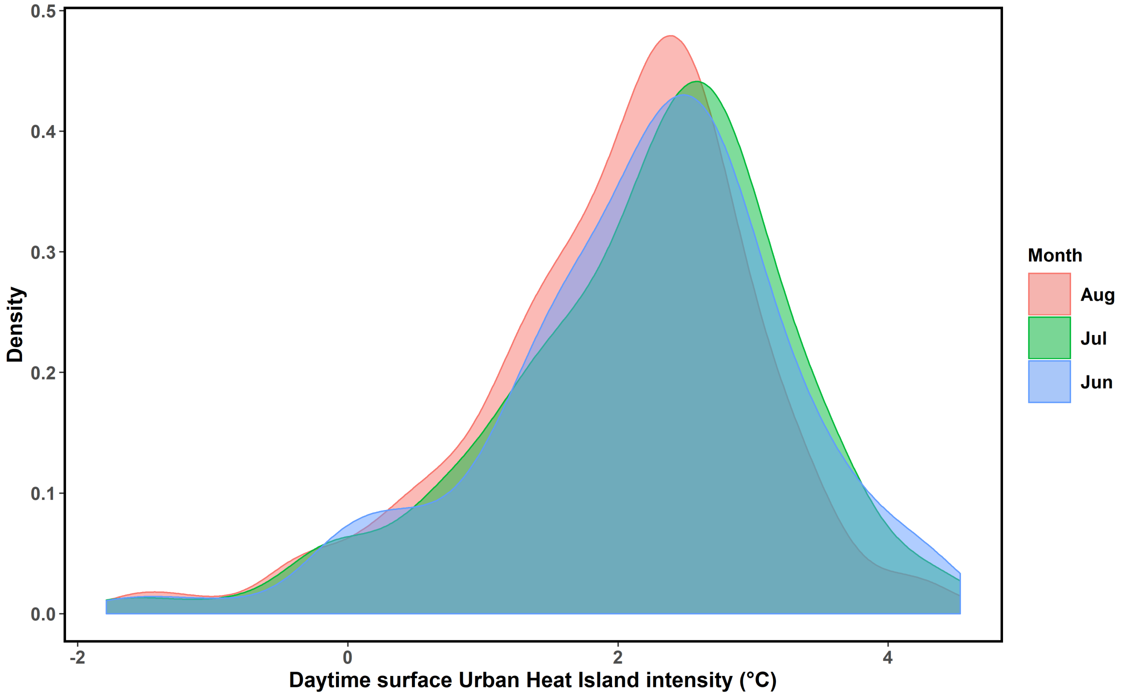

Supplementary Figure 2. Density plots of summer (June, July and August) surface urban heat island intensity in the 175 urbanized areas evaluated in this study

City	POC[a]-White[b]	Below poverty - Above 2×poverty	POC[a] - Below poverty	White[b] - Below poverty	Above 65 - Below 65
**Arid**					
Albuquerque	0.30*	0.17	0.03	−0.28*	−0.43***
Bakersfield	0.33	0.45**	−0.16	−0.49**	−0.06
Boise City	0.31	0.03	0.27	−0.04	0.02
Denver	1.03***	0.86***	−0.01	−1.03***	−0.35***
El Paso	0.26	0.04	0.02	−0.24	−0.06
Fresno	0.69***	0.84***	−0.25*	−0.95***	−0.33
Indio	1.06***	0.45*	0.17	−0.88***	−0.62**
Kennewick	0.31	0.18	0.06	−0.25	−0.13
Lancaster	0.15	0.48**	−0.25	−0.40*	−0.05
Laredo	0.59**	0.65***	−0.33	−0.93***	0.39
Las Vegas	0.57***	0.76***	−0.27**	−0.84***	−0.28**
Lubbock	−0.10	−0.02	−0.07	0.02	0.05
McAllen	−0.38**	−0.16	0.04	0.42**	0.21
Phoenix	0.59***	0.67***	−0.12	−0.71***	−0.26***
San Diego	0.79***	0.70***	−0.13	−0.92***	−0.28
Santa Clarita	0.12	−0.05	0.13	0.01	−0.18
Tucson	0.64***	0.56***	−0.01	−0.65***	−0.55***
Victorville	0.36	0.43	−0.12	−0.48*	−0.20
Visalia	−0.09	−0.10	0.03	0.12	0.15
**Snow**					
Akron	1.54***	1.38***	0.27	−1.27***	−0.30
Albany	2.03***	2.11***	−0.06	−2.09***	−0.45
Allentown	2.43***	1.79***	0.42	−2.01***	−0.32
Ann Arbor	0.24	0.94***	−0.49	−0.73**	−0.32
Appleton	0.77**	0.66**	0.17	−0.60*	0.10
Boston	2.60***	2.16***	0.11	−2.49***	−0.56***
Buffalo	2.17***	1.46***	0.60**	−1.57***	−0.21
Canton	1.92***	1.94***	0.18	−1.74***	−0.18
Chicago	1.40***	0.84***	0.12	−1.27***	−0.20**
Cleveland	1.98***	1.88***	−0.02	−2.00***	−0.26
Columbus	1.18***	1.72***	−0.45**	−1.63***	−0.27
Davenport	0.44	0.30	0.16	−0.29	−0.05
Des Moines	0.55**	0.43*	0.11	−0.44*	0.04
Detroit	1.46***	1.65***	−0.25**	−1.71***	−0.13
Flint	1.61***	1.64***	0.05	−1.56***	−0.26
Fort Collins	−0.10	−0.11	0.02	0.11	−0.03
Fort Wayne	1.07***	1.29***	−0.13	−1.20***	−0.15
Grand Rapids	2.07***	1.69***	0.33	−1.74***	−0.35
Green Bay	0.96**	0.93**	0.09	−0.87**	0.04
Hartford	2.85***	2.55***	−0.17	−3.03***	−0.31
Indianapolis	1.14***	1.57***	−0.36*	−1.50***	−0.17
Kalamazoo	1.30***	1.67***	−0.15	−1.45***	−0.45
Lansing	1.04***	1.43***	−0.16	−1.20***	−0.53
Lincoln	0.53**	0.84***	−0.14	−0.67***	−0.23
Madison	0.24	−0.27	0.40	0.16	0.04
Milwaukee	2.39***	2.00***	0.13	−2.26***	−0.53***
Minneapolis	1.39***	1.55***	−0.23	−1.61***	−0.23*
Nashua	1.89***	1.89**	0.03	−1.85**	0.05
Omaha	0.60***	0.83***	−0.17	−0.77***	−0.14
Peoria	1.18***	1.07***	0.11	−1.07***	−0.11
Portland	0.95**	1.19***	−0.12	−1.07***	−0.28
Poughkeepsie	1.02***	0.92***	−0.01	−1.03***	−0.15
Provo	0.52*	1.02***	−0.35	−0.86***	−0.15

Supplementary Table 1. City differences in surface urban heat island intensity means by race/ethnicity, income, and age. [a]People of color (POC) includes all who do not report as non-Hispanic white alone.[b]Non-Hispanic white alone. $^{*}p < 0.10$, $^{**}p < 0.05$, $^{***}p < 0.01$.

Continued on next page

– *Continued from previous page*

City	POC[a]-White[b]	Below poverty - Above 2×poverty	POC[a] - Below poverty	White[b] - Below poverty	Above 65 - Below 65
Rochester	2.80^{***}	2.58^{***}	0.06	-2.73^{***}	-0.52^{*}
Rockford	0.81^{**}	1.14^{***}	−0.24	-1.06^{***}	−0.09
Round Lake Beach	0.84^{***}	−0.04	0.69^{**}	−0.14	−0.14
Salt Lake City	0.74^{***}	0.73^{***}	−0.01	-0.76^{***}	−0.19
Scranton	2.29^{***}	1.67^{***}	0.80	-1.49^{***}	−0.25
South Bend	1.33^{***}	1.29^{***}	0.12	-1.20^{***}	−0.51
Springfield	2.35^{***}	1.89^{***}	0.27	-2.08^{***}	−0.39
Syracuse	2.90^{***}	2.55^{***}	0.44	-2.46^{***}	−0.39
Toledo	1.30^{***}	1.11^{***}	0.18	-1.12^{***}	−0.23
Worcester	2.65^{***}	2.56^{***}	0.03	-2.62^{***}	−0.37
Youngstown	1.21^{***}	1.13^{***}	0.18	-1.03^{***}	−0.19
**Temperate**					
Antioch	−0.65	−0.62	0.12	0.76	0.30
Asheville	0.70^{**}	0.40	0.29	−0.41	−0.23
Atlanta	1.09^{***}	0.78^{***}	−0.05	-1.14^{***}	-0.37^{***}
Atlantic City	0.98^{***}	0.77^{**}	−0.02	-1.00^{***}	−0.22
Augusta	0.97^{***}	0.85^{***}	−0.05	-1.02^{***}	−0.00
Austin	0.66^{***}	0.74^{***}	−0.20	-0.86^{***}	-0.40^{**}
Baltimore	1.73^{***}	2.05^{***}	-0.71^{***}	-2.44^{***}	-0.37^{**}
Barnstable Town	0.59^{*}	0.45	0.17	−0.42	−0.08
Baton Rouge	0.99^{***}	0.30	0.33	-0.66^{*}	0.27
Birmingham	2.23^{***}	1.79^{***}	−0.00	-2.23^{***}	−0.15
Bremerton	0.73	0.89^{*}	−0.06	−0.78	−0.42
Bridgeport	3.25^{***}	2.62^{***}	−0.05	-3.30^{***}	-0.71^{**}
Cape Coral	0.64^{**}	0.40	0.18	-0.46^{*}	-0.58^{**}
Charleston	0.77^{***}	0.97^{***}	−0.23	-1.01^{***}	−0.24
Charlotte	1.11^{***}	0.92^{***}	−0.07	-1.18^{***}	-0.37^{**}
Chattanooga	1.97^{***}	1.54^{***}	0.40	-1.57^{***}	−0.33
Cincinnati	1.45^{***}	0.96^{***}	0.39^{**}	-1.06^{***}	−0.06
Colorado Springs	0.93^{***}	0.88^{***}	0.00	-0.93^{***}	−0.41
Columbia	0.54^{*}	0.78^{**}	−0.18	-0.72^{**}	−0.29
Columbus	1.36^{**}	1.62^{***}	−0.45	-1.81^{***}	0.03
Concord	0.62^{*}	0.73^{**}	−0.27	-0.89^{***}	−0.54
Concord	0.73^{***}	0.15	0.38	−0.35	−0.15
Conroe	0.53	−0.15	0.50	−0.03	−0.23
Corpus Christi	1.17^{***}	0.28	0.16	-1.00^{**}	0.05
Dallas	0.49^{***}	0.49^{***}	−0.13	-0.62^{***}	−0.11
Danbury	2.43^{***}	2.11^{**}	0.00	-2.43^{***}	−0.50
Dayton	0.45^{*}	0.81^{***}	−0.22	-0.66^{***}	−0.18
Denton	0.58^{*}	0.52	−0.01	-0.59^{*}	−0.26
Durham	1.18^{***}	1.24^{***}	−0.25	-1.43^{***}	−0.58
Eugene	0.62	1.24^{**}	−0.32	-0.94^{**}	−0.60
Evansville	1.11^{**}	1.07^{**}	0.23	-0.88^{**}	−0.05
Fayetteville	0.27	0.20	0.05	−0.22	0.13
Fayetteville	1.18^{***}	0.88^{**}	0.26	-0.93^{***}	-0.86^{**}
Greensboro	1.72^{***}	1.88^{***}	−0.29	-2.01^{***}	−0.45
Greenville	1.07^{***}	0.14	0.67^{**}	−0.41	−0.06
Gulfport	0.52^{*}	0.32	0.16	−0.36	0.06
Hagerstown	0.80	1.27^{***}	−0.40	-1.20^{**}	−0.03
Harrisburg	1.06^{***}	0.64^{*}	0.34	-0.72^{**}	0.05
Hickory	0.82^{***}	0.30	0.45	−0.36	−0.00
Houston	1.07^{***}	0.78^{***}	−0.20	-1.27^{***}	−0.15
Huntington	1.68^{***}	0.69^{*}	1.08^{*}	−0.60	−0.07
Huntsville	0.85^{**}	1.45^{***}	−0.58	-1.43^{***}	−0.12

Supplementary Table 1. City differences in surface urban heat island intensity means by race/ethnicity, income, and age. [a]People of color (POC) includes all who do not report as non-Hispanic white alone.[b]Non-Hispanic white alone. $^{*}p < 0.10$, $^{**}p < 0.05$, $^{***}p < 0.01$.

Continued on next page

– *Continued from previous page*

City	POC[a]-White[b]	Below poverty - Above 2×poverty	POC[a] - Below poverty	White[b] - Below poverty	Above 65 - Below 65
Jackson	0.74**	0.95***	−0.29	−1.04***	−0.07
Jacksonville	1.00***	1.12***	−0.23	−1.23***	−0.20
Kansas City	0.49***	0.51***	−0.06	−0.55***	−0.03
Killeen	0.59	0.25	0.17	−0.42	−0.34
Kissimmee	0.88	0.39	0.03	−0.84	−0.14
Knoxville	1.15***	0.90***	0.32	−0.83**	−0.43*
Lafayette	0.01	0.07	−0.02	−0.03	0.11
Lakeland	0.23	0.10	0.08	−0.15	0.01
Lancaster	2.43***	1.27***	1.02*	−1.42***	−0.20
Lexington	0.37	1.44***	−0.72**	−1.08***	−0.24
Little Rock	1.10***	0.74***	0.14	−0.96***	−0.03
Los Angeles	1.82***	0.77***	0.05	−1.77***	−0.46***
Louisville	1.53***	1.39***	0.11	−1.42***	−0.10
Memphis	0.91***	0.77***	−0.12	−1.03***	−0.06
Mission Viejo	0.64*	−0.03	0.46	−0.18	−0.19
Mobile	0.83**	0.36	0.22	−0.61*	0.04
Modesto	0.51	0.53	−0.08	−0.59	−0.12
Montgomery	1.05**	0.34	0.22	−0.83	0.13
Murrieta	0.29	0.40	−0.18	−0.47	0.40
Myrtle Beach	0.25	0.41	−0.11	−0.36	−0.18
Nashville	0.88***	1.23***	−0.32	−1.19***	−0.30
New Haven	2.34***	2.23***	−0.18	−2.52***	−0.68**
New Orleans	−0.09	0.30	−0.24	−0.15	0.24
New York	2.19***	1.31***	0.03	−2.16***	−0.39***
Norwich	1.00***	1.21***	−0.22	−1.21***	−0.26
Ogden	0.80***	0.84***	0.00	−0.80***	−0.21
Oklahoma City	0.62***	0.70***	−0.09	−0.71***	−0.23
Orlando	0.97***	0.80***	−0.08	−1.04***	−0.09
Oxnard	1.03***	0.52*	0.01	−1.03***	−0.35
Palm Bay	0.39	0.51*	−0.07	−0.47	−0.20
Palm Coast	1.19***	0.73**	0.42	−0.77***	−0.51*
Pensacola	1.00***	0.62*	0.27	−0.74**	−0.16
Philadelphia	2.51***	2.59***	−0.45**	−2.96***	−0.40***
Pittsburgh	1.41***	1.13***	0.31*	−1.10***	−0.22
Portland	0.97***	0.97***	−0.00	−0.98***	−0.39**
Port St. Lucie	1.14***	0.59	0.32	−0.82**	−0.92**
Providence	3.57***	2.31***	0.97***	−2.60***	−0.66**
Raleigh	0.71***	0.80***	−0.17	−0.88***	−0.23
Reading	3.32***	2.79***	0.20	−3.11***	−0.62
Reno	0.59**	0.50**	0.04	−0.55**	−0.40
Richmond	0.97***	1.39***	−0.51**	−1.48***	−0.29
Riverside	0.97***	0.88***	−0.31**	−1.28***	−0.25*
Roanoke	2.19***	1.59***	0.50	−1.69***	−0.35
Sacramento	0.49***	0.55***	−0.15	−0.63***	−0.29*
St. Louis	1.23***	0.93***	0.15	−1.08***	−0.06
Salem	1.38***	1.47***	−0.01	−1.38***	−0.62
San Antonio	0.86***	0.81***	−0.27**	−1.13***	−0.04
San Francisco	1.51***	0.53**	0.17	−1.34***	−0.58**
San Jose	0.86***	0.51***	−0.12	−0.98***	−0.24
Santa Rosa	0.88***	0.42	0.24	−0.63*	−0.80**
Sarasota	0.70**	0.87***	−0.10	−0.79***	−0.31
Savannah	1.32***	1.58***	−0.39	−1.72***	−0.13
Seattle	1.01***	1.21***	−0.29*	−1.30***	−0.40**
Shreveport	0.90**	0.88**	−0.11	−1.01**	−0.10
Spokane	0.50*	0.86***	−0.10	−0.60**	−0.22

Supplementary Table 1. City differences in surface urban heat island intensity means by race/ethnicity, income, and age. [a]People of color (POC) includes all who do not report as non-Hispanic white alone.[b]Non-Hispanic white alone. $^{*}p < 0.10$, $^{**}p < 0.05$, $^{***}p < 0.01$.

Continued on next page

– *Continued from previous page*

City	POC[a]-White[b]	Below poverty - Above 2×poverty	POC[a] - Below poverty	White[b] - Below poverty	Above 65 - Below 65
Springfield	0.96**	1.45***	−0.07	−1.04***	−0.24
Stockton	0.84**	0.90***	−0.31	−1.15***	−0.31
Tallahassee	0.79	2.55***	−1.20**	−1.99***	−1.11**
Tampa	0.48***	0.75***	−0.21	−0.69***	0.01
Trenton	2.04***	1.82***	−0.36	−2.40***	−0.62
Tulsa	0.62**	0.60**	−0.00	−0.62**	−0.11
Virginia Beach	1.22***	1.00***	−0.10	−1.32***	−0.28
Washington, D.C.	0.73***	0.94***	−0.46***	−1.19***	−0.28***
Wichita	0.61***	0.86***	−0.20	−0.81***	−0.08
Wilmington	0.77*	0.96**	−0.06	−0.83*	−0.37
Winston	1.36***	1.15***	0.09	−1.26***	−0.23
Winter Haven	0.71**	0.37	0.18	−0.53*	−0.28
York	2.95***	2.83***	0.09	−2.86***	−0.52
**Equatorial**					
Bonita Springs	0.26	0.42	−0.16	−0.42	−0.07
Miami	1.25***	1.01***	−0.25**	−1.50***	−0.17

Supplementary Table 1. City differences in surface urban heat island intensity means by race/ethnicity, income, and age. [a]People of color (POC) includes all who do not report as non-Hispanic white alone.[b]Non-Hispanic white alone. $^{*}p < 0.10, ^{**}p < 0.05, ^{***}p < 0.01$.

		People per tract								
					Non-Hispanic			Poverty Status		
City	Tracts	Total	POC	Hispanic	Black	White	Other	<1×	1-2×	>2×
Akron	147	4061	802	72	512	3259	217	586	735	2644
		(1707)	(830)	(76)	(706)	(1782)	(259)	(444)	(425)	(1596)
Albany	166	4019	951	224	371	3068	356	455	513	2896
		(1757)	(833)	(191)	(524)	(1708)	(309)	(406)	(330)	(1701)
Albuquerque	173	4569	2733	2216	112	1837	405	785	877	2855
		(1995)	(1660)	(1531)	(132)	(1098)	(409)	(588)	(554)	(1544)
Allentown	167	4662	1205	749	228	3456	228	508	709	3286
		(1769)	(1191)	(953)	(236)	(1759)	(222)	(514)	(454)	(1658)
Ann Arbor	89	3575	1167	165	486	2408	515	551	450	2399
		(1448)	(707)	(129)	(512)	(1270)	(473)	(674)	(292)	(1318)
Antioch	45	6324	4187	2259	871	2137	1057	819	1115	4348
		(2623)	(1871)	(1111)	(661)	(1535)	(750)	(553)	(672)	(2301)
Appleton	58	4398	512	201	58	3886	253	375	622	3334
		(2085)	(329)	(153)	(80)	(2006)	(190)	(249)	(318)	(1877)
Asheville	95	4472	673	310	211	3799	151	592	908	2877
		(1671)	(496)	(319)	(284)	(1515)	(142)	(305)	(517)	(1257)
Atlanta	885	6019	3229	657	2057	2790	514	814	1049	4062
		(3010)	(2460)	(815)	(2194)	(2307)	(614)	(634)	(782)	(2495)
Atlantic City	76	3657	1517	621	510	2140	387	533	613	2435
		(2497)	(1604)	(680)	(726)	(1783)	(566)	(483)	(482)	(2054)
Augusta	94	5225	2357	288	1811	2868	259	874	1001	3220
		(2726)	(1710)	(326)	(1521)	(2132)	(271)	(594)	(572)	(2357)
Austin	302	5726	2731	1822	404	2995	506	655	834	4144
		(3124)	(2159)	(1687)	(509)	(1964)	(567)	(697)	(691)	(2755)
Bakersfield	95	5996	3902	3113	322	2094	467	1311	1362	3266
		(3453)	(2819)	(2348)	(357)	(1811)	(637)	(1096)	(1019)	(2744)
Baltimore	576	4006	1952	241	1349	2054	362	448	533	2918
		(1893)	(1499)	(336)	(1362)	(1635)	(439)	(367)	(392)	(1778)
Barnstable Town	64	4077	407	108	99	3670	199	302	492	3227
		(1495)	(339)	(121)	(132)	(1376)	(199)	(190)	(274)	(1343)
Baton Rouge	127	5651	2457	232	1981	3194	244	905	905	3722
		(2882)	(1517)	(281)	(1493)	(2885)	(256)	(720)	(508)	(2592)
Birmingham	209	4246	1784	184	1450	2463	150	635	727	2811
		(2116)	(1282)	(247)	(1276)	(2220)	(171)	(453)	(451)	(1969)
Boise City	59	7375	1090	580	92	6285	418	855	1131	5249
		(5013)	(636)	(428)	(132)	(4584)	(274)	(613)	(722)	(4159)
Bonita Springs	76	5007	1587	1207	264	3421	116	532	853	3575
		(3799)	(2109)	(1731)	(407)	(2401)	(130)	(543)	(906)	(2789)
Boston	985	4787	1383	518	360	3404	505	464	520	3646
		(1743)	(1250)	(768)	(672)	(1847)	(476)	(420)	(382)	(1679)
Bremerton	53	4724	1081	354	121	3642	607	458	636	3491
		(1611)	(628)	(249)	(125)	(1269)	(363)	(267)	(381)	(1477)
Bridgeport	205	4399	1629	840	478	2770	311	388	552	3378
		(1605)	(1410)	(837)	(625)	(1661)	(270)	(353)	(470)	(1516)
Buffalo	264	3913	908	193	497	3005	219	573	603	2647
		(1677)	(981)	(263)	(824)	(1943)	(268)	(489)	(292)	(1718)
Canton	80	4471	580	82	305	3891	192	586	771	3019
		(1744)	(504)	(86)	(413)	(1782)	(137)	(431)	(368)	(1643)
Cape Coral	154	4199	1358	859	365	2840	135	630	856	2645
		(2583)	(1465)	(991)	(604)	(1862)	(127)	(578)	(698)	(1742)
Charleston	141	4887	1720	277	1215	3167	228	637	802	3327
		(3034)	(1193)	(315)	(951)	(2603)	(201)	(465)	(520)	(2649)
Charlotte	323	4629	1980	534	1114	2650	332	557	758	3254
		(1988)	(1544)	(554)	(1078)	(1893)	(339)	(472)	(543)	(1866)
Chattanooga	100	4562	1107	215	713	3455	178	645	850	2944
		(2112)	(1063)	(285)	(955)	(2153)	(170)	(502)	(443)	(1843)
Chicago	2070	4288	2076	961	747	2212	368	554	683	2980

Supplementary Table 2. City demographic summary statistics. Standard deviations in parentheses. People of color (POC) includes all who do not report as non-Hispanic white alone.

Continued on next page

– Continued from previous page

		People per tract								
					Non-Hispanic			Poverty Status		
City	Tracts	Total	POC	Hispanic	Black	White	Other	<1×	1-2×	>2×
		(2164)	(1535)	(1218)	(1145)	(1962)	(496)	(459)	(519)	(1947)
Cincinnati	417	4308	970	138	607	3338	224	547	630	3042
		(2358)	(965)	(194)	(840)	(2337)	(305)	(447)	(378)	(2247)
Cleveland	582	3225	985	154	676	2240	155	459	509	2198
		(1717)	(814)	(237)	(803)	(1894)	(171)	(377)	(310)	(1665)
Colorado Springs	129	5171	1564	855	302	3607	406	555	844	3629
		(2372)	(1059)	(618)	(280)	(1819)	(288)	(448)	(553)	(2264)
Columbia	158	4324	1889	229	1441	2435	220	608	754	2745
		(2583)	(1776)	(269)	(1558)	(1736)	(220)	(456)	(454)	(2203)
Columbus	74	4119	2145	275	1626	1975	244	772	851	2326
		(2616)	(1516)	(329)	(1378)	(2015)	(282)	(446)	(536)	(2156)
Columbus	340	4639	1457	216	858	3182	383	650	696	3195
		(2483)	(1257)	(223)	(1021)	(2178)	(412)	(577)	(484)	(2347)
Concord	132	5538	2371	778	155	3167	1438	324	419	4731
		(1810)	(1518)	(756)	(207)	(1318)	(1228)	(306)	(416)	(1738)
Concord	70	5052	1667	480	953	3385	234	683	939	3348
		(2267)	(1110)	(362)	(753)	(1944)	(309)	(415)	(501)	(1969)
Conroe	49	9104	3019	2042	517	6084	460	799	1301	6934
		(5890)	(2177)	(1508)	(547)	(4270)	(458)	(663)	(873)	(5429)
Corpus Christi	82	4388	2974	2677	160	1413	138	656	899	2734
		(1681)	(1394)	(1350)	(153)	(1208)	(153)	(490)	(556)	(1493)
Dallas	1122	5334	3003	1614	891	2331	498	724	1012	3540
		(2772)	(2003)	(1395)	(1088)	(2022)	(655)	(618)	(742)	(2503)
Danbury	50	5099	1335	821	161	3764	353	338	530	4105
		(1716)	(1298)	(963)	(177)	(1434)	(331)	(352)	(394)	(1464)
Davenport	87	3684	845	341	317	2839	187	476	595	2516
		(1462)	(483)	(268)	(313)	(1435)	(147)	(294)	(238)	(1366)
Dayton	206	3889	910	110	589	2979	210	578	664	2532
		(1985)	(842)	(132)	(818)	(2010)	(230)	(414)	(430)	(1849)
Denton	74	5112	1919	1056	389	3193	475	526	681	3746
		(2339)	(1055)	(864)	(316)	(1960)	(285)	(521)	(546)	(2179)
Denver	581	4582	1683	1094	258	2899	331	475	678	3371
		(1800)	(1388)	(1097)	(437)	(1495)	(265)	(436)	(547)	(1588)
Des Moines	109	4917	994	369	277	3924	348	515	710	3608
		(3215)	(759)	(341)	(302)	(3123)	(307)	(389)	(444)	(2942)
Detroit	1158	3312	1199	146	818	2113	236	537	542	2199
		(1581)	(1024)	(328)	(995)	(1700)	(340)	(480)	(352)	(1496)
Durham	85	5049	2501	622	1448	2549	431	763	792	3218
		(2074)	(1680)	(583)	(1269)	(1618)	(399)	(597)	(602)	(1960)
El Paso	166	5232	4591	4320	153	640	118	1153	1393	2594
		(3216)	(2923)	(2769)	(255)	(666)	(159)	(701)	(819)	(2388)
Eugene	66	4410	836	400	54	3574	382	821	873	2612
		(1428)	(508)	(337)	(63)	(1086)	(276)	(728)	(394)	(1140)
Evansville	68	4112	572	97	306	3540	170	643	726	2617
		(2409)	(458)	(127)	(307)	(2307)	(179)	(385)	(456)	(2176)
Fayetteville	80	4999	2781	583	1731	2218	467	880	1113	2790
		(2687)	(1572)	(394)	(1110)	(1484)	(338)	(497)	(618)	(2031)
Fayetteville	70	6054	1663	1022	160	4391	482	812	1257	3846
		(2117)	(1564)	(1215)	(213)	(1633)	(461)	(705)	(860)	(1583)
Flint	128	3206	883	105	638	2322	140	627	616	1924
		(1548)	(822)	(95)	(773)	(1623)	(140)	(490)	(373)	(1352)
Fort Collins	73	4547	780	522	40	3766	218	533	643	3240
		(2625)	(526)	(416)	(47)	(2244)	(180)	(439)	(461)	(2316)
Fort Wayne	95	3868	981	278	436	2887	268	559	748	2494
		(1481)	(923)	(268)	(562)	(1593)	(261)	(439)	(414)	(1537)
Fresno	154	4675	3116	2109	282	1559	725	1135	979	2497
		(1748)	(1612)	(1138)	(311)	(1083)	(553)	(843)	(590)	(1538)

Supplementary Table 2. City demographic summary statistics. Standard deviations in parentheses. People of color (POC) includes all who do not report as non-Hispanic white alone.

Continued on next page

– Continued from previous page

		People per tract								
					Non-Hispanic			Poverty Status		
City	Tracts	Total	POC	Hispanic	Black	White	Other	<1×	1-2×	>2×
Grand Rapids	137	5116	1241	498	438	3875	305	658	888	3460
		(2001)	(1045)	(652)	(601)	(2102)	(230)	(574)	(504)	(1898)
Green Bay	53	4880	897	404	106	3983	387	535	848	3358
		(2731)	(732)	(476)	(125)	(2522)	(399)	(342)	(550)	(2439)
Greensboro	77	4392	2193	338	1573	2200	282	751	888	2594
		(1939)	(1595)	(321)	(1337)	(1692)	(259)	(622)	(556)	(1682)
Greenville	127	4419	1216	353	684	3204	179	691	927	2679
		(2056)	(864)	(353)	(616)	(1899)	(200)	(512)	(478)	(1713)
Gulfport	57	4908	1604	271	1031	3303	302	884	1021	2894
		(3136)	(1038)	(226)	(793)	(2536)	(283)	(617)	(714)	(2165)
Hagerstown	46	5934	1059	268	514	4875	277	713	984	4021
		(2654)	(835)	(191)	(613)	(2403)	(196)	(484)	(519)	(2232)
Harrisburg	121	4619	1038	277	472	3581	289	437	666	3378
		(1862)	(982)	(340)	(633)	(2001)	(263)	(386)	(327)	(1769)
Hartford	239	4165	1456	666	484	2709	305	443	531	3048
		(1613)	(1135)	(745)	(672)	(1833)	(345)	(398)	(385)	(1645)
Hickory	61	5004	1004	386	342	4000	277	810	1142	2930
		(1494)	(693)	(363)	(346)	(1413)	(250)	(356)	(450)	(1161)
Houston	908	6236	4170	2411	1122	2066	637	938	1186	4046
		(4421)	(3217)	(1927)	(1415)	(2381)	(1130)	(740)	(877)	(3885)
Huntington	74	3695	246	43	102	3449	100	700	692	2196
		(1692)	(230)	(68)	(162)	(1698)	(88)	(393)	(342)	(1386)
Huntsville	72	5103	1792	242	1233	3311	317	656	711	3578
		(3204)	(1277)	(246)	(1055)	(2687)	(300)	(560)	(488)	(2929)
Indianapolis	323	5215	1551	372	872	3663	308	705	881	3543
		(3215)	(1344)	(439)	(999)	(3029)	(411)	(553)	(549)	(2986)
Indio	92	4302	2552	2255	94	1750	202	796	1028	2456
		(2192)	(2394)	(2389)	(133)	(1194)	(187)	(739)	(820)	(1304)
Jackson	101	4481	2340	91	2141	2141	107	703	812	2834
		(2757)	(1595)	(130)	(1599)	(2367)	(126)	(519)	(553)	(2474)
Jacksonville	215	5618	2272	500	1348	3346	425	775	985	3749
		(3552)	(1788)	(495)	(1430)	(2688)	(502)	(558)	(640)	(3064)
Kalamazoo	56	4716	1046	236	491	3670	318	753	794	3013
		(2059)	(756)	(193)	(590)	(1952)	(223)	(682)	(435)	(1862)
Kansas City	439	3780	1160	381	536	2620	243	446	615	2670
		(2075)	(824)	(418)	(654)	(2014)	(219)	(386)	(413)	(1956)
Kennewick	46	5523	2030	1618	91	3493	320	759	1051	3661
		(2745)	(2023)	(1957)	(101)	(2001)	(276)	(780)	(882)	(2039)
Killeen	51	5055	2911	1133	1261	2144	517	637	1145	3016
		(3311)	(2334)	(861)	(1204)	(1504)	(405)	(460)	(854)	(2537)
Kissimmee	43	8442	5768	4460	772	2674	535	1363	2312	4710
		(5498)	(4660)	(3598)	(981)	(1894)	(489)	(982)	(1819)	(3223)
Knoxville	171	4283	616	171	273	3667	171	632	769	2791
		(1703)	(568)	(190)	(445)	(1707)	(148)	(551)	(439)	(1471)
Lafayette	64	5546	1942	237	1475	3603	230	968	1065	3399
		(2571)	(1143)	(189)	(1166)	(2511)	(217)	(531)	(502)	(2245)
Lakeland	68	4171	1460	687	591	2711	182	645	935	2512
		(1821)	(883)	(537)	(594)	(1425)	(137)	(492)	(480)	(1508)
Lancaster	101	5404	938	539	192	4466	207	544	895	3830
		(1889)	(932)	(655)	(245)	(1994)	(162)	(376)	(405)	(1672)
Lancaster	74	4963	3479	2369	760	1484	350	994	1166	2683
		(2365)	(1861)	(1464)	(546)	(1089)	(283)	(663)	(673)	(1856)
Lansing	102	3603	1008	263	372	2595	373	629	578	2209
		(1439)	(705)	(180)	(404)	(1322)	(363)	(573)	(342)	(1402)
Laredo	60	4439	4283	4237	12	156	34	1365	1172	1844
		(2566)	(2405)	(2333)	(42)	(220)	(79)	(747)	(633)	(1969)
Las Vegas	469	4387	2489	1364	482	1898	643	637	899	2803

Supplementary Table 2. City demographic summary statistics. Standard deviations in parentheses. People of color (POC) includes all who do not report as non-Hispanic white alone.

Continued on next page

– *Continued from previous page*

		People per tract								
					Non-Hispanic			Poverty Status		
City	Tracts	Total	POC	Hispanic	Black	White	Other	<1×	1-2×	>2×
		(1757)	(1323)	(953)	(415)	(1158)	(535)	(470)	(529)	(1514)
Lexington	87	3894	1050	267	523	2844	260	666	634	2439
		(1538)	(846)	(367)	(561)	(1398)	(233)	(687)	(380)	(1514)
Lincoln	72	4079	757	285	165	3322	307	562	666	2667
		(1656)	(565)	(262)	(171)	(1497)	(254)	(481)	(481)	(1508)
Little Rock	120	4453	1697	252	1241	2756	204	671	860	2858
		(2079)	(1275)	(267)	(1145)	(2058)	(197)	(490)	(480)	(1736)
Los Angeles	2788	4557	3290	2130	292	1267	867	720	918	2850
		(1828)	(1773)	(1563)	(537)	(1244)	(979)	(543)	(608)	(1547)
Louisville	265	4117	1056	190	662	3061	204	537	673	2814
		(1604)	(968)	(206)	(869)	(1613)	(188)	(442)	(400)	(1539)
Lubbock	63	4476	2051	1544	314	2425	193	820	884	2579
		(2282)	(1417)	(1157)	(414)	(1633)	(183)	(618)	(579)	(1802)
McAllen	111	7445	6948	6828	32	496	89	2341	1960	3045
		(3197)	(3106)	(3074)	(92)	(480)	(176)	(1512)	(965)	(1903)
Madison	98	4831	1015	318	254	3816	442	614	574	3514
		(2246)	(832)	(392)	(272)	(1987)	(385)	(749)	(412)	(2212)
Memphis	269	4264	2517	246	2092	1747	179	810	822	2557
		(2424)	(1807)	(406)	(1699)	(1952)	(254)	(668)	(533)	(2257)
Miami	1206	4961	3396	2199	992	1565	205	783	1069	3050
		(2358)	(2418)	(2082)	(1421)	(1349)	(248)	(663)	(740)	(1794)
Milwaukee	407	3615	1251	395	631	2364	225	535	578	2431
		(1518)	(1068)	(613)	(930)	(1801)	(202)	(425)	(337)	(1589)
Minneapolis	684	4328	1156	272	400	3173	484	421	566	3271
		(2081)	(968)	(303)	(490)	(1900)	(462)	(430)	(398)	(1960)
Mission Viejo	109	5230	1801	986	78	3429	737	337	490	4383
		(2002)	(1089)	(859)	(89)	(1381)	(453)	(279)	(432)	(1805)
Mobile	109	3605	1565	99	1313	2040	153	688	725	2105
		(2366)	(928)	(134)	(921)	(2179)	(186)	(405)	(467)	(1857)
Modesto	76	5514	3097	2439	136	2417	521	944	1304	3210
		(2322)	(1707)	(1375)	(138)	(1376)	(590)	(511)	(633)	(1970)
Montgomery	77	3935	2149	136	1856	1786	157	737	748	2306
		(2230)	(1461)	(241)	(1395)	(1914)	(224)	(631)	(446)	(1688)
Murrieta	76	6474	3305	2182	334	3168	789	643	988	4795
		(2721)	(1752)	(1195)	(292)	(1361)	(657)	(437)	(543)	(2362)
Myrtle Beach	77	4225	921	255	523	3304	142	663	901	2604
		(2166)	(809)	(328)	(616)	(1773)	(135)	(552)	(544)	(1478)
Nashua	51	5131	679	301	78	4452	301	328	527	4221
		(2001)	(670)	(463)	(87)	(1849)	(274)	(390)	(428)	(1817)
Nashville	253	4575	1526	384	879	3049	263	578	734	3169
		(1975)	(1236)	(438)	(974)	(1795)	(248)	(483)	(533)	(1796)
New Haven	151	4590	1462	627	543	3129	292	441	556	3427
		(1794)	(1358)	(746)	(796)	(1837)	(235)	(441)	(408)	(1686)
New Orleans	342	2854	1573	278	1146	1281	149	555	536	1709
		(1654)	(1317)	(372)	(1170)	(1179)	(234)	(446)	(378)	(1247)
New York	4469	4316	2341	1057	694	1975	590	592	647	3002
		(2061)	(1874)	(1237)	(1058)	(1778)	(762)	(639)	(554)	(1715)
Norwich	66	4074	969	412	212	3105	345	398	523	2969
		(1624)	(889)	(496)	(281)	(1415)	(282)	(402)	(385)	(1388)
Ogden	110	5448	995	684	61	4454	249	470	867	4062
		(2543)	(667)	(598)	(75)	(2373)	(172)	(333)	(476)	(2371)
Oklahoma City	291	3478	1351	515	423	2128	413	503	655	2259
		(1961)	(880)	(642)	(507)	(1623)	(307)	(437)	(453)	(1731)
Omaha	225	3607	917	398	302	2689	218	407	579	2546
		(1357)	(798)	(574)	(450)	(1368)	(186)	(355)	(389)	(1378)
Orlando	299	5966	3058	1564	1032	2908	462	890	1201	3750
		(4063)	(2464)	(1506)	(1273)	(2412)	(522)	(700)	(921)	(3151)

Supplementary Table 2. City demographic summary statistics. Standard deviations in parentheses. People of color (POC) includes all who do not report as non-Hispanic white alone.

Continued on next page

– *Continued from previous page*

		People per tract								
					Non-Hispanic			Poverty Status		
City	Tracts	Total	POC	Hispanic	Black	White	Other	<1×	1-2×	>2×
Oxnard	83	5028	3363	2843	102	1666	418	665	1068	3226
		(2488)	(2421)	(2169)	(121)	(1395)	(432)	(529)	(731)	(1922)
Palm Bay	95	5192	1318	539	500	3874	279	663	997	3469
		(2849)	(1186)	(510)	(657)	(2084)	(253)	(527)	(746)	(2080)
Palm Coast	92	4378	993	307	480	3385	206	673	914	2730
		(1975)	(950)	(279)	(740)	(1684)	(194)	(456)	(514)	(1501)
Pensacola	85	4742	1458	253	841	3285	364	636	876	2958
		(2112)	(890)	(233)	(745)	(1904)	(285)	(408)	(483)	(1734)
Peoria	75	4113	836	150	454	3277	233	514	670	2815
		(1771)	(820)	(140)	(633)	(1857)	(311)	(343)	(352)	(1697)
Philadelphia	1410	4137	1570	377	853	2567	341	534	573	2933
		(1754)	(1487)	(632)	(1171)	(1726)	(377)	(589)	(451)	(1598)
Phoenix	893	4562	1956	1362	222	2606	371	716	841	2956
		(1941)	(1587)	(1364)	(263)	(1611)	(386)	(670)	(607)	(1637)
Pittsburgh	599	3282	516	56	297	2766	163	361	471	2371
		(1715)	(543)	(60)	(463)	(1737)	(208)	(289)	(263)	(1599)
Portland	63	4390	439	96	136	3951	206	466	588	3221
		(1704)	(374)	(103)	(206)	(1631)	(162)	(337)	(375)	(1551)
Portland	431	4863	1322	578	148	3541	596	594	753	3453
		(1845)	(930)	(545)	(212)	(1350)	(535)	(474)	(481)	(1535)
Port St. Lucie	68	6056	2116	990	910	3941	215	881	1249	3863
		(4129)	(2336)	(1149)	(1213)	(2561)	(276)	(730)	(942)	(3007)
Poughkeepsie	119	4489	1385	710	420	3104	256	490	562	3227
		(1586)	(953)	(581)	(461)	(1534)	(198)	(612)	(401)	(1528)
Providence	281	4414	1114	595	229	3301	290	569	665	3029
		(1653)	(1254)	(915)	(295)	(1768)	(241)	(468)	(433)	(1551)
Provo	123	4515	771	511	23	3743	237	523	848	3029
		(1885)	(571)	(447)	(36)	(1643)	(177)	(569)	(497)	(1648)
Raleigh	206	5575	2117	550	1076	3458	491	547	787	4131
		(2820)	(1922)	(581)	(1340)	(1956)	(620)	(538)	(685)	(2349)
Reading	68	4405	1489	1144	205	2916	140	689	770	2833
		(2116)	(1088)	(987)	(182)	(2434)	(128)	(544)	(359)	(2203)
Reno	105	4172	1511	1000	90	2661	421	550	794	2778
		(1564)	(1011)	(834)	(95)	(1240)	(280)	(442)	(538)	(1350)
Richmond	255	4306	1905	274	1317	2401	314	540	620	3044
		(1992)	(1460)	(367)	(1273)	(1682)	(426)	(508)	(429)	(1904)
Riverside	370	5528	4080	3121	450	1448	508	956	1212	3251
		(2429)	(2093)	(1720)	(423)	(1210)	(584)	(691)	(679)	(2110)
Roanoke	56	4624	1058	185	644	3566	230	617	821	3059
		(1504)	(1091)	(197)	(960)	(1581)	(231)	(502)	(378)	(1356)
Rochester	206	3940	1073	309	531	2867	233	536	592	2704
		(1822)	(816)	(298)	(566)	(2010)	(265)	(386)	(322)	(1815)
Rockford	76	3922	1263	586	477	2660	199	611	820	2423
		(2038)	(813)	(541)	(480)	(1886)	(141)	(424)	(488)	(1841)
Round Lake Beach	57	5267	1187	786	117	4079	284	421	705	4120
		(1699)	(1019)	(825)	(135)	(1580)	(282)	(231)	(437)	(1437)
Sacramento	396	4813	2344	995	378	2470	970	703	821	3231
		(1932)	(1675)	(705)	(415)	(1359)	(879)	(583)	(589)	(1667)
St. Louis	510	4499	1357	141	973	3142	243	544	667	3194
		(1893)	(1272)	(176)	(1259)	(2123)	(235)	(444)	(420)	(1799)
Salem	45	5920	1946	1418	74	3973	455	899	1308	3513
		(2345)	(1461)	(1210)	(107)	(1802)	(283)	(616)	(767)	(1832)
Salt Lake City	213	5233	1465	938	86	3768	440	541	887	3738
		(2730)	(1213)	(931)	(133)	(2289)	(334)	(451)	(600)	(2388)
San Antonio	397	5253	3586	2975	361	1667	250	798	1068	3296
		(2269)	(1830)	(1693)	(443)	(1450)	(292)	(580)	(702)	(2034)
San Diego	610	5251	2849	1763	252	2402	834	686	890	3545

Supplementary Table 2. City demographic summary statistics. Standard deviations in parentheses. People of color (POC) includes all who do not report as non-Hispanic white alone.

Continued on next page

– *Continued from previous page*

		People per tract								
					Non-Hispanic			Poverty Status		
City	Tracts	Total	POC	Hispanic	Black	White	Other	<1×	1-2×	>2×
		(2890)	(2194)	(1541)	(347)	(1850)	(1012)	(562)	(755)	(2172)
San Francisco	789	4526	2863	1008	348	1663	1507	484	594	3377
		(1767)	(1731)	(1025)	(467)	(1164)	(1187)	(428)	(490)	(1528)
San Jose	337	5183	3533	1294	127	1650	2112	439	595	4073
		(1669)	(1681)	(1120)	(123)	(1108)	(1349)	(351)	(487)	(1501)
Santa Clarita	61	4434	2293	1406	182	2140	705	357	486	3460
		(1980)	(1436)	(1150)	(191)	(1062)	(492)	(410)	(519)	(1662)
Santa Rosa	67	5364	2095	1522	89	3270	483	583	878	3828
		(2081)	(1569)	(1266)	(107)	(1257)	(332)	(379)	(628)	(1531)
Sarasota	174	4249	914	520	254	3335	139	492	761	2940
		(1835)	(1007)	(597)	(503)	(1658)	(129)	(424)	(454)	(1549)
Savannah	78	4189	1998	267	1511	2191	220	656	714	2650
		(3446)	(1793)	(387)	(1378)	(2261)	(293)	(429)	(497)	(2927)
Scranton	138	3217	422	196	112	2795	114	476	577	2029
		(1334)	(436)	(225)	(179)	(1334)	(145)	(314)	(275)	(1114)
Seattle	660	5217	1906	516	301	3311	1089	541	667	3930
		(1678)	(1171)	(440)	(367)	(1323)	(736)	(421)	(459)	(1543)
Shreveport	80	4443	2131	185	1779	2312	167	913	883	2547
		(2617)	(1517)	(229)	(1498)	(2423)	(168)	(653)	(522)	(2197)
South Bend	83	3552	953	289	464	2599	200	615	696	2093
		(1847)	(612)	(319)	(376)	(1769)	(194)	(384)	(375)	(1546)
Spokane	97	4661	703	251	76	3958	376	676	884	2971
		(2013)	(452)	(158)	(96)	(1815)	(277)	(459)	(468)	(1741)
Springfield	163	4519	1332	792	303	3187	237	631	672	2937
		(1864)	(1230)	(905)	(456)	(1915)	(255)	(594)	(473)	(1654)
Springfield	71	4764	531	166	136	4233	230	797	992	2803
		(1760)	(326)	(125)	(152)	(1666)	(171)	(467)	(403)	(1701)
Stockton	84	4988	3769	2206	450	1219	1114	1030	1189	2663
		(3026)	(2429)	(1221)	(370)	(1010)	(1413)	(524)	(661)	(2486)
Syracuse	141	3436	762	155	357	2674	250	506	515	2280
		(1471)	(643)	(142)	(448)	(1627)	(264)	(394)	(274)	(1465)
Tallahassee	71	4224	1835	254	1336	2389	246	821	730	2494
		(1939)	(1040)	(204)	(936)	(1577)	(227)	(922)	(468)	(1867)
Tampa	655	4074	1526	778	493	2547	256	582	800	2630
		(1854)	(1324)	(824)	(692)	(1346)	(274)	(451)	(512)	(1513)
Toledo	156	3653	923	223	532	2730	168	600	637	2343
		(1776)	(682)	(193)	(569)	(1829)	(153)	(425)	(346)	(1696)
Trenton	63	4318	2194	820	1095	2124	279	558	687	2882
		(1729)	(1301)	(788)	(962)	(1810)	(285)	(440)	(471)	(1712)
Tucson	213	4187	2012	1574	135	2175	303	776	863	2436
		(1787)	(1489)	(1393)	(157)	(1362)	(267)	(641)	(584)	(1382)
Tulsa	212	3640	1290	403	331	2350	556	522	684	2381
		(1862)	(929)	(508)	(498)	(1495)	(365)	(412)	(434)	(1612)
Victorville	51	7174	4669	3557	718	2505	395	1569	1624	3794
		(3752)	(3248)	(2294)	(734)	(1361)	(419)	(1009)	(836)	(2875)
Virginia Beach	375	4044	1895	272	1294	2148	330	485	625	2766
		(2036)	(1294)	(264)	(1083)	(1546)	(290)	(402)	(414)	(1698)
Visalia	43	5877	3683	3223	111	2193	350	1269	1391	3131
		(3486)	(2412)	(2054)	(103)	(1576)	(416)	(986)	(645)	(2579)
Washington, D.C.	1150	4512	2582	748	1168	1930	666	370	490	3574
		(1827)	(1573)	(816)	(1244)	(1411)	(650)	(348)	(438)	(1640)
Wichita	124	4211	1308	588	363	2903	356	600	792	2763
		(2229)	(942)	(554)	(492)	(2079)	(368)	(420)	(476)	(2095)
Wilmington	55	5009	1127	271	670	3882	187	850	802	3229
		(2636)	(824)	(275)	(657)	(2316)	(171)	(658)	(575)	(2013)
Winston	118	4199	1511	456	877	2688	178	712	801	2584
		(1611)	(1218)	(442)	(883)	(1632)	(183)	(538)	(469)	(1466)

Supplementary Table 2. City demographic summary statistics. Standard deviations in parentheses. People of color (POC) includes all who do not report as non-Hispanic white alone.

Continued on next page

– *Continued from previous page*

		People per tract								
					Non-Hispanic			Poverty Status		
City	Tracts	Total	POC	Hispanic	Black	White	Other	<1×	1-2×	>2×
Winter Haven	64	4199	1650	870	662	2549	118	824	1042	2294
		(1945)	(1274)	(835)	(736)	(1275)	(102)	(543)	(560)	(1271)
Worcester	118	4758	1136	523	246	3622	367	535	615	3432
		(1928)	(979)	(599)	(280)	(1920)	(429)	(507)	(382)	(1873)
York	56	4865	1031	452	371	3834	209	570	794	3346
		(2262)	(741)	(414)	(305)	(2328)	(176)	(415)	(450)	(2061)
Youngstown	131	3407	663	129	424	2743	110	589	644	2087
		(1670)	(635)	(158)	(522)	(1791)	(93)	(394)	(312)	(1517)

Supplementary Table 2. City demographic summary statistics. Standard deviations in parentheses. People of color (POC) includes all who do not report as non-Hispanic white alone.

Supplementary Note 1

As discussed in detail in Sheriff and Maguire (8), the KP inequality index has several properties that make it more useful in the context of SUHI evaluation than the Atkinson inequality index. The KP index allows the outcome variable to take negative values. This feature makes it well-suited for examining distributions of SUHI intensity, since, unlike other environmental stressors (e.g., air pollution), the SUHI can have negative values at the local scale. That is, a census tract can be cooler than its city's rural reference.

Moreover, unlike the Atkinson index the KP index is insensitive to an additive shift in all outcomes by the same constant. This feature implies that ranking of heat for demographic groups within a given urbanized area is insensitive to whether it is measured in raw temperatures or SUHI intensity (raw temperature minus a common rural reference).

The inequality aversion parameter κ in Eq. (2) reflects the non-linearity of damages caused by summer heat exposure. Specifically, the elasticity of marginal utility to a change in SUHI is κx_n. A lower value of κ corresponds to a higher marginal damage of x, and a higher inequality index value for a given unequal distribution. At the limit as κ approaches zero, the KP index approaches zero and the EDE approaches the mean.

Apart from κ, Eq. (2) reflects several assumptions about the agent's preferences: (i) all else equal, increasing any x_n is undesirable; (ii) preferences across x_n are Schur-concave, i.e., all else equal, shifting an amount Δ UHI from an individual x_i to x_j is desirable if $x_i - x_j > \Delta$;[1] and (iii) preferences are *translation invariant* (1), i.e., a ranking of lotteries will not change if every outcome in each lottery is shifted by an additive constant. The first two assumptions are fairly uncontroversial.

Translation invariance, however, represents an important distinction from the Atkinson inequality index which assumes scale invariance (lottery rankings do not change if each outcome is multiplied by a positive constant). Scale invariance is a convenient property for comparing income distributions in different points of time or space since it eliminates the need for adjusting for inflation or exchange rates. It is less justifiable for evaluating distributions

[1]Formally, let $\mathbf{Q}$ be a square matrix composed of non-negative real numbers whose rows and columns each sum to 1. The function $f(\mathbf{x})$ is Schur concave if $\mathbf{Qx}$ is not a permutation of $\mathbf{x}$ and $f(\mathbf{Qx}) \geq f(\mathbf{x})$. All symmetric quasiconcave functions are Schur concave, although the converse is not true (5).

of environmental inequality, however. Doubling all SUHI exposures, for example, would double the gap in exposures between any two individuals yet would not change the inequality measured by the Atkinson index.

In general, distributional rankings may be sensitive to the elasticity of marginal utility specified by the value of the inequality aversion parameter. Since there is not a consensus regarding the "right" value, the literature typically presents results for a range of values. In the context of income distribution, the U.S. Census Bureau has reported results with elasticities of 0.25, 0.5, and 0.75 (7, 6). For environmental outcomes (3) evaluated elasticities ranging from 0.25 to 2.0 in their study of the distribution of outdoor NO_2 and Cropper et al. (4) estimated a mean value of 0.72.

These studies use Atkinson inequality measures for which the elasticity is a constant. For KP measures, however, this elasticity, κx_n, is a function of x. To present results for a range of κ that generates elasticities comparable to those in the above-cited literature, we first identify a value of κ that is consistent with a given constant elasticity β. To establish a correspondence between an elasticity β and a vector of elasticities $\kappa\mathbf{x}$, we use the approach of Sheriff and Maguire (8), choosing the value of κ that minimizes the sum of squared differences between the individual elasticities and β:

$$\begin{aligned} \kappa(\beta) &= -\arg\min_{\hat{\kappa}} \{[\hat{\kappa}\mathbf{x} - \beta\mathbf{1}]'[\hat{\kappa}\mathbf{x} - \beta\mathbf{1}]\} \\ &= -\frac{\beta \sum_{n=1}^{N} x_n}{\sum_{n=1}^{N} x_n^2}. \end{aligned} \tag{1}$$

We use $\kappa(0.50)$, representing "moderate" inequality aversion to calculate the main KP index results presented in Table 2. Tables 3–5 display present EDE results for low ($\kappa(0.25)$), moderate ($\kappa(0.50)$), and high ($\kappa(0.75)$) levels of inequality aversion. Although EDE and index magnitudes vary with different inequality aversion parameter values, the qualitative results remain largely unchanged.

	Climate zone				
	Arid	Snow	Temperate	Equatorial	Total
a. Population-weighted EDE means					
Total	0.46	2.37	2.36	2.87	2.20
	(0.45)	(0.89)	(1.23)	(0.43)	(1.23)
By race/ethnicity[a]					
People of color	0.70	3.56	3.07	3.29	2.89
	(0.51)	(1.06)	(1.45)	(0.35)	(1.51)
Hispanic	0.79	3.77	3.15	3.13	2.81
	(0.52)	(1.26)	(1.48)	(0.35)	(1.60)
Non-Hispanic					
Black	0.78	3.81	3.16	3.82	3.23
	(0.65)	(0.99)	(1.62)	(0.35)	(1.55)
White	0.18	1.80	1.69	2.02	1.61
	(0.45)	(0.74)	(0.98)	(0.24)	(0.97)
Other	0.28	2.81	2.77	2.44	2.56
	(0.47)	(1.02)	(1.31)	(0.17)	(1.39)
By income					
Below poverty	0.80	3.45	3.06	3.51	2.90
	(0.51)	(1.09)	(1.46)	(0.45)	(1.50)
Above 2 × poverty	0.29	2.01	2.11	2.52	1.94
	(0.46)	(0.86)	(1.19)	(0.36)	(1.18)
b. Difference in mean EDE values					
People of color − Non-Hisp. white	0.52^{***}	1.76^{***}	1.38^{***}	1.27^{**}	1.28^{***}
	(0.172)	(0.272)	(0.465)	(0.137)	(0.355)
Below poverty − 2 × poverty	0.51^{***}	1.44^{***}	0.95^{*}	0.99^{**}	0.96^{***}
	(0.162)	(0.304)	(0.489)	(0.181)	(0.363)
People of color − below poverty	-0.10	0.12	0.01	-0.22	-0.01
	(0.166)	(0.297)	(0.553)	(0.151)	(0.419)
Non-Hisp. white − below poverty	-0.61^{***}	-1.64^{***}	-1.37^{***}	-1.49^{**}	-1.29^{***}
	(0.167)	(0.267)	(0.434)	(0.166)	(0.318)
c. Proportion of urban areas for which					
People of color > Non-Hisp. white	0.84	0.98	0.98	1.00	0.97
Below poverty > 2 × poverty	0.79	0.93	0.97	1.00	0.94
People of color > below poverty	0.42	0.61	0.42	0.00	0.46
Non-Hisp. white > below poverty	0.16	0.05	0.01	0.00	0.03

Supplementary Table 3. Low inequality aversion equally distributed equivalent (EDE) summer daytime surface urban heat island intensity by climate. People of color refers to all individuals who do not report as non-Hispanic white alone. Panel a: Population-weighted mean of urban area Kolm-Pollak EDEs in °C. Standard deviation in parentheses. Panel b: Robust standard errors in parentheses. [a]People of color includes all who do not identify as non-Hispanic white alone. Hispanic includes all reporting this ethnicity, regardless of race. Black and white include all non-Hispanics identifying as these races alone. Other includes all other races alone and more than one race. $^{*}p < 0.10, ^{**}p < 0.05, ^{***}p < 0.01$.
Source: Author calculations, based on data from American Community Survey and (2).

	Climate zone				
	Arid	Snow	Temperate	Equatorial	Total
a. Population-weighted EDE means					
Total	0.52	2.52	2.50	2.96	2.33
	(0.45)	(0.93)	(1.27)	(0.45)	(1.27)
By race/ethnicity					
People of color	0.75	3.68	3.18	3.38	3.00
	(0.51)	(1.09)	(1.48)	(0.36)	(1.54)
Hispanic	0.83	3.90	3.25	3.22	2.90
	(0.52)	(1.30)	(1.51)	(0.36)	(1.63)
Non-Hispanic					
Black	0.83	3.90	3.27	3.89	3.33
	(0.66)	(1.00)	(1.63)	(0.35)	(1.57)
White	0.25	1.94	1.82	2.11	1.74
	(0.44)	(0.78)	(1.01)	(0.25)	(1.01)
Other	0.34	2.93	2.90	2.54	2.68
	(0.46)	(1.06)	(1.32)	(0.18)	(1.42)
By income					
Below poverty	0.84	3.57	3.17	3.59	3.01
	(0.52)	(1.13)	(1.49)	(0.46)	(1.53)
Above 2 × poverty	0.35	2.15	2.24	2.62	2.07
	(0.45)	(0.90)	(1.23)	(0.38)	(1.22)
b. Difference in mean EDE values					
People of color − Non-Hisp. white	0.50^{***}	1.74^{***}	1.36^{***}	1.27^{**}	1.26^{***}
	(0.165)	(0.283)	(0.473)	(0.145)	(0.362)
Below poverty − 2 × poverty	0.49^{***}	1.42^{***}	0.93^{*}	0.97^{**}	0.94^{**}
	(0.159)	(0.314)	(0.498)	(0.188)	(0.370)
People of color − below poverty	-0.10	0.11	0.01	-0.21	-0.01
	(0.165)	(0.306)	(0.558)	(0.154)	(0.424)
Non-Hisp. white − below poverty	-0.60^{***}	-1.63^{***}	-1.35^{***}	-1.48^{**}	-1.27^{***}
	(0.161)	(0.277)	(0.441)	(0.174)	(0.324)
c. Proportion of urban areas for which					
People of color > Non-Hisp. white	0.84	0.98	0.97	1.00	0.96
Below poverty > 2 × poverty	0.79	0.93	0.98	1.00	0.95
People of color > below poverty	0.42	0.57	0.41	0.00	0.45
Non-Hisp. white > below poverty	0.16	0.05	0.01	0.00	0.03

Supplementary Table 4. Moderate inequality aversion equally distributed equivalent (EDE) summer daytime surface urban heat island intensity by climate. People of color refers to all individuals who do not report as non-Hispanic white alone. Panel a: Population-weighted mean of urban area Kolm-Pollak EDEs in °C. Standard deviation in parentheses. Panel b: Robust standard errors in parentheses. [a]People of color includes all who do not identify as non-Hispanic white alone. Hispanic includes all reporting this ethnicity, regardless of race. Black and white include all non-Hispanics identifying as these races alone. Other includes all other races alone and more than one race. $^{*}p < 0.10,^{**} p < 0.05,^{***} p < 0.01$.
Source: Author calculations, based on data from American Community Survey and (2).

	Climate zone				
	Arid	Snow	Temperate	Equatorial	Total
a. Population-weighted EDE means					
Total	0.58	2.66	2.63	3.06	2.46
	(0.45)	(0.98)	(1.31)	(0.46)	(1.31)
By race/ethnicity					
People of color	0.80	3.79	3.29	3.47	3.10
	(0.51)	(1.12)	(1.50)	(0.37)	(1.57)
Hispanic	0.88	4.01	3.35	3.31	2.99
	(0.52)	(1.33)	(1.54)	(0.37)	(1.66)
Non-Hispanic					
Black	0.87	3.99	3.37	3.96	3.43
	(0.67)	(1.01)	(1.65)	(0.35)	(1.58)
White	0.32	2.08	1.96	2.19	1.86
	(0.44)	(0.83)	(1.05)	(0.27)	(1.05)
Other	0.40	3.06	3.03	2.63	2.81
	(0.46)	(1.11)	(1.34)	(0.19)	(1.45)
By income					
Below poverty	0.89	3.69	3.29	3.66	3.11
	(0.52)	(1.16)	(1.52)	(0.47)	(1.57)
Above 2 × poverty	0.41	2.28	2.37	2.72	2.19
	(0.45)	(0.94)	(1.27)	(0.40)	(1.26)
b. Difference in mean EDE values					
People of color − Non-Hisp. white	0.48^{***}	1.71^{***}	1.34^{***}	1.27^{**}	1.24^{***}
	(0.162)	(0.295)	(0.479)	(0.153)	(0.368)
Below poverty − 2 × poverty	0.48^{***}	1.40^{***}	0.92^{*}	0.95^{**}	0.92^{**}
	(0.159)	(0.324)	(0.505)	(0.194)	(0.377)
People of color − below poverty	-0.10	0.10	0.00	-0.20	-0.01
	(0.167)	(0.315)	(0.562)	(0.157)	(0.428)
Non-Hisp. white − below poverty	-0.58^{***}	-1.61^{***}	-1.33^{***}	-1.47^{**}	-1.25^{***}
	(0.159)	(0.287)	(0.447)	(0.181)	(0.330)
c. Proportion of urban areas for which					
People of color > Non-Hisp. white	0.84	0.98	0.97	1.00	0.96
Below poverty > 2 × poverty	0.79	0.93	0.98	1.00	0.95
People of color > below poverty	0.42	0.55	0.42	0.00	0.45
Non-Hisp. white > below poverty	0.16	0.05	0.01	0.00	0.03

Supplementary Table 5. High inequality aversion equally distributed equivalent (EDE) summer daytime surface urban heat island intensity by climate. People of color refers to all individuals who do not report as non-Hispanic white alone. Panel a: Population-weighted mean of urban area Kolm-Pollak EDEs in °C. Standard deviation in parentheses. Panel b: Robust standard errors in parentheses. [a]People of color includes all who do not identify as non-Hispanic white alone. Hispanic includes all reporting this ethnicity, regardless of race. Black and white include all non-Hispanics identifying as these races alone. Other includes all other races alone and more than one race. $^{*}p < 0.10, ^{**}p < 0.05, ^{***}p < 0.01$.
Source: Author calculations, based on data from American Community Survey and Chakraborty et al. (2).

Supplementary References

[1] Charles Blackorby and David Donaldson. A theoretical treatment of indices of absolute inequality. *International Economic Review*, 21(1):107–136, February 1980.

[2] T Chakraborty, A Hsu, D Manya, and G Sheriff. A spatially explicit surface urban heat island database for the United States: Characterization, uncertainties, and possible applications. *ISPRS Journal of Photogrammetry and Remote Sensing*, 168:74–88, 2020.

[3] Lara P Clark, Dylan B Millet, and Julian D Marshall. National patterns in environmental injustice and inequality: Outdoor NO_2 air pollution in the United States. *PloS one*, 9 (4):e94431, 2014.

[4] Maureen Cropper, Alan Krupnick, and William Reich. Preferences for equality in environmental outcomes. *NBER Working Paper No. 22644*, 2016.

[5] Partha Dasgupta, Amartya Sen, and David Starrett. Notes on the measurement of inequality. *Journal of Economic Theory*, 6(2):180–187, 1973.

[6] Carmen DeNavas-Walt, Bernadette D. Proctor, and Jessica C. Smith. Income, poverty, and health insurance coverage in the United States: 2011. In *Current Population Report*, number P60-243, pages 1–81. U.S. Census Bureau, 2012.

[7] Arthur F. Jones, Jr. and Daniel H. Weinberg. The changing shape of the nation's income distribution 1947–1998. In *Current Population Reports*, number P60-204, pages 1–11. U.S. Census Bureau, 2000.

[8] Glenn Sheriff and Kelly Maguire. Health risk, inequality indexes, and environmental justice. *Risk Analysis*, page doi.org/10.1111/risa.13562, 2020.
