# Supplementary material for: Disproportionate exposure to urban heat island intensity across major US cities
Source: Nat Commun. 2021 May 25;12:2721. doi: 10.1038/s41467-021-22799-5 (PMC8149665; doi:10.1038/s41467-021-22799-5)
Supplement: Supplementary file 3 — Reporting Summary [file 41467_2021_22799_MOESM3_ESM.pdf]

nature research

Corresponding author(s): Angel Hsu

Last updated by author(s): 11/16/2020

Reporting Summary

Nature Research wishes to improve the reproducibility of the work that we publish. This form provides structure for consistency and transparency in reporting. For further information on Nature Research policies, see our Editorial Policies and the Editorial Policy Checklist.

Statistics

For all statistical analyses, confirm that the following items are present in the figure legend, table legend, main text, or Methods section.

n/a	Confirmed	
☐	☒	The exact sample size (*n*) for each experimental group/condition, given as a discrete number and unit of measurement
☐	☒	A statement on whether measurements were taken from distinct samples or whether the same sample was measured repeatedly
☐	☒	The statistical test(s) used AND whether they are one- or two-sided<br>*Only common tests should be described solely by name; describe more complex techniques in the Methods section.*
☐	☒	A description of all covariates tested
☐	☒	A description of any assumptions or corrections, such as tests of normality and adjustment for multiple comparisons
☐	☒	A full description of the statistical parameters including central tendency (e.g. means) or other basic estimates (e.g. regression coefficient) AND variation (e.g. standard deviation) or associated estimates of uncertainty (e.g. confidence intervals)
☐	☒	For null hypothesis testing, the test statistic (e.g. *F*, *t*, *r*) with confidence intervals, effect sizes, degrees of freedom and *P* value noted<br>*Give P values as exact values whenever suitable.*
☒	☐	For Bayesian analysis, information on the choice of priors and Markov chain Monte Carlo settings
☒	☐	For hierarchical and complex designs, identification of the appropriate level for tests and full reporting of outcomes
☒	☐	Estimates of effect sizes (e.g. Cohen's *d*, Pearson's *r*), indicating how they were calculated

Our web collection on statistics for biologists contains articles on many of the points above.

Software and code

Policy information about availability of computer code

Data collection	SUHI intensity data are available for exploration on an interactive Google Earth Engine platform tool, available at https://datadrivenlab.users.earthengine.app/view/usuhiapp and for download at https://data.mendeley.com/datasets/x9mv4krnm2/2. Sociodemographic data were collected from the US Census Bureau 2017 5-year American Community Survey via the API at https://api.census.gov/data/2017/acs/acs5/variables.html.
Data analysis	All statistical analyses were conducted in STATA (Version 15) and R (Version 3.6.3).

For manuscripts utilizing custom algorithms or software that are central to the research but not yet described in published literature, software must be made available to editors and reviewers. We strongly encourage code deposition in a community repository (e.g. GitHub). See the Nature Research guidelines for submitting code & software for further information.

Data

Policy information about availability of data

All manuscripts must include a data availability statement. This statement should provide the following information, where applicable:

- Accession codes, unique identifiers, or web links for publicly available datasets
- A list of figures that have associated raw data
- A description of any restrictions on data availability

SUHI intensity data are available for exploration on an interactive Google Earth Engine platform tool, available at https://datadrivenlab.users.earthengine.app/view/usuhiapp and also at https://data.mendeley.com/datasets/x9mv4krnm2/2. Sociodemographic data were collected from the US Census Bureau 2017 5-year American Community Survey via the API at https://api.census.gov/data/2017/acs/acs5/variables.html.

Field-specific reporting

Please select the one below that is the best fit for your research. If you are not sure, read the appropriate sections before making your selection.

☐ Life sciences ☒ Behavioural & social sciences ☐ Ecological, evolutionary & environmental sciences

For a reference copy of the document with all sections, see nature.com/documents/nr-reporting-summary-flat.pdf

Behavioural & social sciences study design

All studies must disclose on these points even when the disclosure is negative.

Study description	We combine US-specific surface urban heat island (SUHI) data, a proxy for isolating the urban contribution to additional heat exposure in built environments, with census tract-level demographic data from the American Community Survey to conduct a quantitative study utilizing statistical analysis methods (i.e., difference of means and inequality indexes) and to evaluates whether certain populations within major U.S. cities are exposed to higher levels of SUHI.
Research sample	All census tracts within U.S. cities with a population above 250,000 as measured by the 2017 5-year American Community Survey (n=175).
Sampling strategy	We selected all census tracts within U.S. cities with a population above 250,000 as measured by the 2017 5-year American Community Survey.
Data collection	All authors were involved in data collection (Hsu, Sheriff, Chakraborty, and Hsu) and were blind to the study hypothesis during the data collection process. Specifically, Chakraborty downloaded and compiled the SUHI intensity data was from satellite-remote sensing measurements of land-surface temperature (see Chakraborty et al., 2020 for details of how we developed the dataset). Hsu and Sheriff downloaded sociodemographic data for all census tracts within U.S. cities with a population above 250,000 from the US Census Bureau's website for the 2017 5-year American Community Survey and their API in R Studio. Manya and Hsu developed a series of functions in R to merge the SUHI and US Census data in R Studio. Sheriff independently verified all data and calculations in STATA.
Timing	We began data collection for the SUHI data on August 19, 2019 and completed it October 29, 2019. We began collection of the US Census Bureau data on September 1, 2019 and completed it August 4, 2020.
Data exclusions	We excluded cities with populations lower than 250,000 (n=302), although the SUHI intensity data developed for this study is fully available for all 497 urbanised areas in the United States on our Google Earth Engine app. We chose not to include these smaller cities because most of the impacts of heat-related mortality and morbidity are found in major urban metropolitan areas, according to the CDC.
Non-participation	N/A
Randomization	We surveyed all U.S. urban areas.

Reporting for specific materials, systems and methods

We require information from authors about some types of materials, experimental systems and methods used in many studies. Here, indicate whether each material, system or method listed is relevant to your study. If you are not sure if a list item applies to your research, read the appropriate section before selecting a response.

Materials & experimental systems

n/a	Involved in the study	
☒	☐	Antibodies
☒	☐	Eukaryotic cell lines
☒	☐	Palaeontology and archaeology
☒	☐	Animals and other organisms
☒	☐	Human research participants
☒	☐	Clinical data
☒	☐	Dual use research of concern

Methods

n/a	Involved in the study	
☒	☐	ChIP-seq
☒	☐	Flow cytometry
☒	☐	MRI-based neuroimaging
